# Recent Progress on Synthesis of *N*,*N*′-Chelate Organoboron Derivatives

**DOI:** 10.3390/molecules26051401

**Published:** 2021-03-05

**Authors:** Tianbao Yang, Niu Tang, Qizhong Wan, Shuang-Feng Yin, Renhua Qiu

**Affiliations:** State Key Laboratory of Chemo/Biosensing and Chemometrics, Advanced Catalytic Engineering Research Center of the Ministry of Education, College of Chemistry and Chemical Engineering, Institute of Shenzhen, Hunan University, Changsha 410082, China; yangtianbao@hnu.edu.cn (T.Y.); tangniu@hnu.edu.cn (N.T.); sf_yin@hnu.edu.cn (S.-F.Y.)

**Keywords:** organoboron, *N*,*N*′-chelate, tetracoordinated, fluorescent materials

## Abstract

*N*,*N*′-chelate organoboron compounds have been successfully applied in bioimaging, organic light-emitting diodes (OLEDs), functional polymer, photocatalyst, electroluminescent (EL) devices, and other science and technology areas. However, the concise and efficient synthetic methods become more and more significant for material science, biomedical research, or other practical science. Here, we summarized the organoboron-*N*,*N*′-chelate derivatives and showed the different routes of their syntheses. Traditional methods to synthesize *N*,*N*′-chelate organoboron compounds were mainly using bidentate ligand containing nitrogen reacting with trivalent boron reagents. In this review, we described a series of bidentate ligands, such as bipyridine, 2-(pyridin-2-yl)-1*H*-indole, 2-(5-methyl-1*H*-pyrrol-2-yl)quinoline, *N*-(quinolin-8-yl)acetamide, 1,10-phenanthroline, and diketopyrrolopyrrole (DPP).

## 1. Introduction

**Figure 1:**
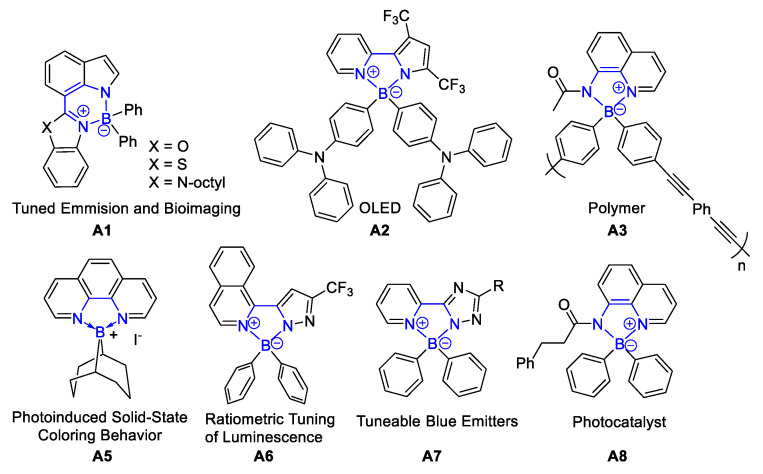
Application of tetracoordinate organoboron complexes in luminescent materials, organic light-emitting diode (OLED), functional polymer, and photocatalyst [30,31,32,33,34,35,36].

*N*,*N*′-chelate tetracoordinated organoboron compounds have been widely applied in various science and technology areas. For example, boron dipyrromethene derivatives (BODIPY) were developed in luminescent materials [1,2,3,4,5], dyes [6,7,8,9], photosensitizers [10,11,12,13,14], molecular switches [15,16,17], photodynamic therapy [18,19,20,21,22], molecular probes [23,24,25,26], and bioimaging [27,28,29]. In recent decades, *N*,*N*′-chelate compounds become a popular topic and have gradually attracted the attention of scientists. Four-coordinated organoboron compounds (BAr_2_ (N, N)) have interesting luminescent properties that can be modulated by various substituents in the *N*, *N*′-chelating framework [30]. More and more similar structures were explored and exhibited wonderful results in fluorescent materials [30,31], cell bioimaging (**A1**) [32], organic light-emitting diodes (OLEDs) (**A2**) [33], functional polymer (**A3**) [34], photocatalyst (**A8**) [35], electroluminescent (EL) devices [36], and so on, as shown in Figure 1. Herein, various traditional methods for the formation of *N*,*N*′-chelate tetracoordinated organoboron complexes were exhibited in this review. The main highlights include that (a) the diversified synthetic methods were provided, (b) readily available trivalent boron compounds were used as boron reagents, (c) various complex starting materials were designed as good bidentate ligands, and (d) these reactions showed a wide range of tetracoordinated organoboron complexes and excellent optical properties. In this paper, we made a conclusion of all articles of the corresponding organic synthetic routes.

**Figure 2:**
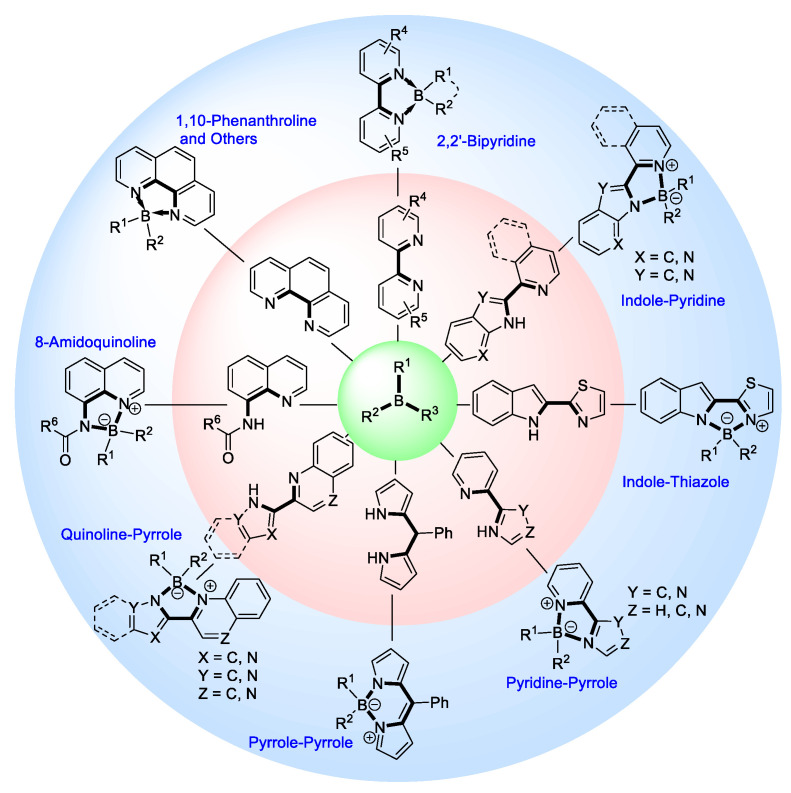
The methods for the formation of *N*,*N*′-chelate organoboron derivatives [30,31,32,33,34,35,36].

In recent years, the research on their syntheses falls into the following categories. At first, bipyridine derivatives could be used as bidentate ligand reacting with triphenylboron to form the desired products, as shown in Figure 2. The second, an indole connecting with pyridine derivatives (or other nitrogen heterocyclic molecule) reacted with triphenylboron to obtain the corresponding compounds, as shown in Figure 2. The third, pyridine attaching on pyrrole derivatives (or other nitrogen heterocyclic molecule) and trivalent boron could produce the fluorescent organoboron compounds, as shown in Figure 2. The fourth, a quinoline linking with pyrrole derivatives (or other nitrogen heterocyclic molecule) has been reported as a bidentate ligand to react with boron reagents. The different fluorescent compounds could be synthesized by *N*-(quinolin-8-yl)acetamide derivatives and boron reagents, as shown in Figure 2. The fifth, other bidentate ligands including 1,10-phenanthroline and diketopyrrolopyrrole (DPP) derivatives could also react with trivalent boron to obtain tetracoordinated organoboron compounds, as shown in Figure 2.

## 2. Bipyridine-Based Derivatives as Bidentate Ligand

In this section, different traditional methods for the formation of *N*,*N*′-chelate organoboron derivatives will be displayed in detail from the following aspects.

### 2.1. Bipyridine as Bidentate Ligand

**Scheme 1:**
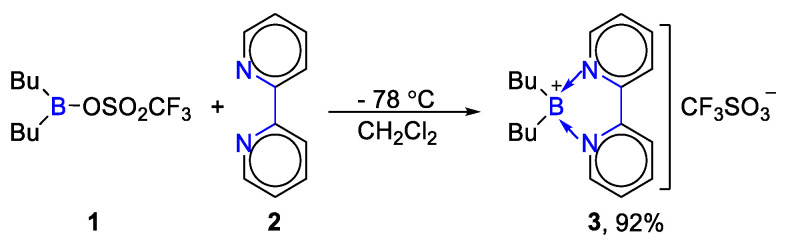
Reactions of bipyridine with dibutyl(((trifluoromethyl)sulfonyl)oxy)borane [37].

In 1985, Heinrich Noeth and co-workers reported that dibutyl(((trifluoromethyl)sulfonyl)oxy)borane (**1**) reacted with bipyridine (**2**) to complete desired product. The solution of the diorganylborane should be cooled to −78 °C in this reaction. Bipyridine-dibutylboronium(1+) triflate (**3**) was confirmed by ^11^B NMR in their lab [37], as shown in Scheme 1. This protocol realized the synthesis of bipyridine coordinated organoboron complex at low temperature. It played a certain role in promoting the deep study of tetracoordinated organoboron compounds.

### 2.2. 2,5-Di(pyridin-2-yl)pyrazine and 2′, 2′: 4′, 4′: 2″, 2‴-Quaterpyridine as Bidentate Ligand

**Scheme 2:**
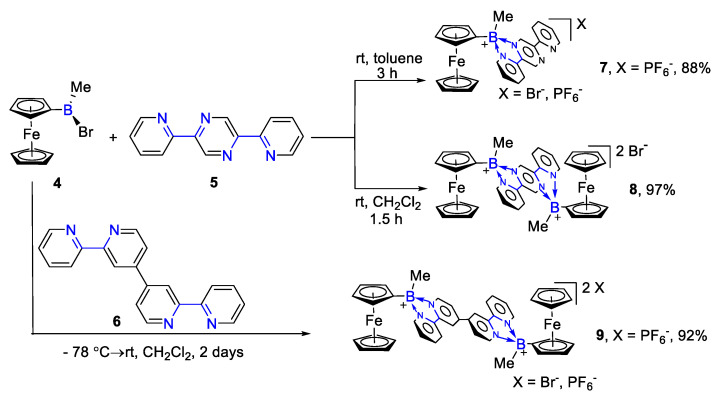
Reactions of 2,5-di(pyridin-2-yl)pyrazine (or 2′, 2′: 4′, 4′: 2″, 2‴-quaterpyridine) with FcB(Me)Br [38].

In 2002, Matthias Wagner’s group adopted ferrocenylboranes (**4**) to react with 2,5-bis(pyridyl)pyrazine (**5**) and 2′, 2′: 4′, 4′: 2″, 2‴-quaterpyridine (**6**) to obtain charge-transfer complexes, respectively [38]. The organoboron adducts possess green color, which is charge transfer from the electron-rich ferrocene skeleton to their electron-poor aromatic structures, as shown in Scheme 2. This strategy provided a new route to acquire more ferrocenylborane derivatives with high yield and simple operation.

### 2.3. Bipyridine in {Fc(Bbipy)_2_O}(PF_6_) and {Fc(Bbipy}_2_(OH)_2_}(PF_6_)

**Scheme 3:**
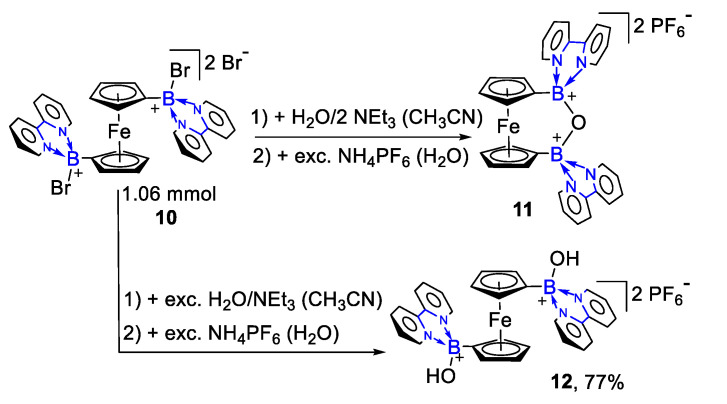
The methods for the synthesis of {Fc(Bbipy)_2_O}(PF_6_)_2_ and {Fc(Bbipy)_2_(OH)_2_}(PF_6_)_2_ [39,40].

In 2002, Matthias Wagner’s group studied the synthesis of ferrocene complexes {Fc(Bbipy)_2_O}(PF_6_)_2_ (**11**) and {Fc(Bbipy)_2_(OH)_2_}(PF_6_)_2_ (**12**) [39,40], as shown in Scheme 3. They successfully synthesized tetracoordinated organoboron compounds with two boron centers. All of these compounds have the property of charge transfer. They could realize ring-closing and ring-opening products using bromide (FcMeBr) for the formation of **11** and **12** by adding different amounts of water in the reaction.

### 2.4. 4,4′-Di(but-3-en-1-yl)-2,2′-bipyridine as Bidentate Ligand

**Scheme 4:**
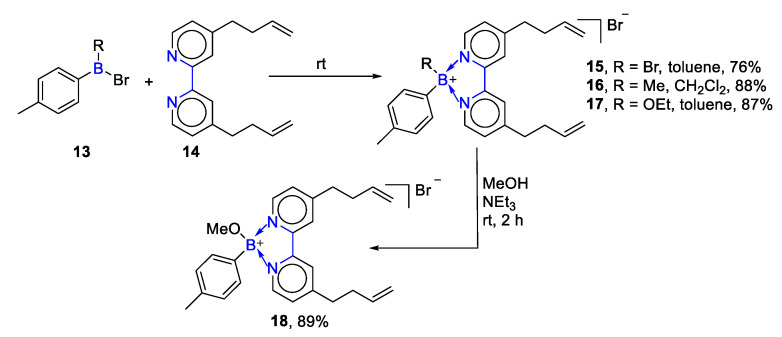
Reactions of 4,4′-di(but-3-en-1-yl)-2,2′-bipyridine with trivalent boron [41].

In 2005, Matthias Wagner’s team reported other *N*,*N*′-chelate organoboron complexes in this reaction. They used dibromo(phenyl)borane, bromo(methyl)(phenyl)borane, bromo(ethoxy)(phenyl)borane to give bipyridine adducts with satisfactory yields (**15**–**18**, 76–89%) [41], as shown in Scheme 4. This reaction indicated that different *N*,*N*′-chelate organoboron compounds could be obtained by adding distinct boron reagents under ambient temperature.

### 2.5. Bipyridine Reacting with 5-Chloro-5,10-dihydrodibenzo[b,e]borinine

**Scheme 5:**
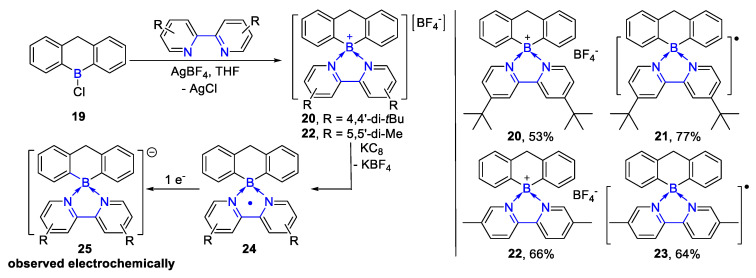
Reactions of bipyridine with 5-chloro-5,10-dihydrodibenzo[b,e]borinine [42].

In 2009, Warren E. Piers’s lab prepared a series of neutral radicals, which had significant spin density on boron [42]. The scaffold of 2,2′-bipyridyl-stabilized boronium ions was interesting and demonstrated bipyridine adducts persistent neutral radical. They added AgBF_4_ in this reaction and offered moderate yields (**20**–**23**, yield 53–77%), as shown in Scheme 5. It was proved that this protocol could easily get the target 2,2′-bipyridyl boronium ions and neutral radicals.

### 2.6. Bipyridine for the Formation of N,N′-Chelate Organoboron and Ferrocene Derivatives

**Scheme 6:**
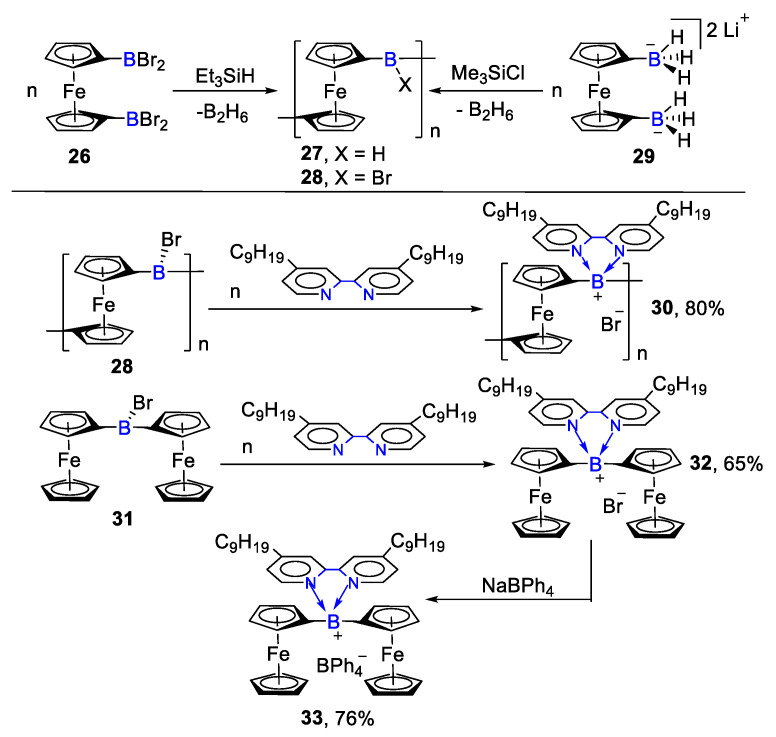
Bipyridine for the formation of *N*,*N*′-chelate organoboron and ferrocene derivatives [43].

In 2010, Matthias Wagner’s group reported a more powerful synthetic route of ferrocene complexes [43]. They used bromide (**26**) to obtain FcBBr polymers (**27**, **28**) and continued to form the bipyridine organoboron polymers with main chain charge-transfer structure (**30**, **32**, **33**, 80%, 65%, 76%), as shown in Scheme 6. This method provided a new opportunity for the synthesis of multifunctional organoboron polymers.

### 2.7. Bipyridine Reacting with 5-Bromo-10-mesityl-5,10-dihydroboranthrene

**Scheme 7:**
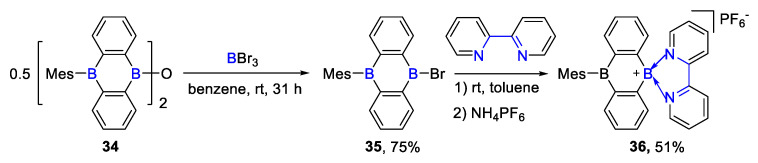
Bipyridine reacting with 5-bromo-10-mesityl-5,10-dihydroboranthrene [44].

In 2011, Matthias Wagner’s group has been committed to the development of more diversified *N*,*N*′-chelate organoboron chemistry for many years [44]. It always showed wonderful results in bipyridine organoboron adducts. They realized cleavage of the B-O-B bridge in this reaction and got the desired product with moderate yield (**36**, 51%), as shown in Scheme 7. In this protocol, they prepared 5-bromo-10-mesityl-5,10-dihydroboranthrene (**35**) as a boron reagent. This building block revealed a novel synthetic route for us.

## 3. Indole-Based Derivatives as Bidentate Ligand

### 3.1. 2-(Pyridin-2-yl)-1H-indole and 2-(Pyridin-2-yl)-1H-pyrrolo[2,3-b]pyridine

**Scheme 8:**
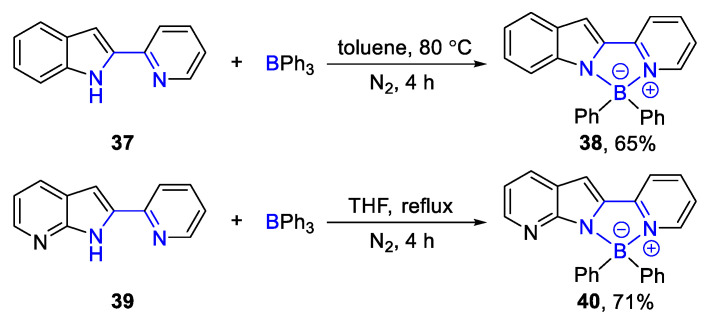
Reactions of 2-(pyridin-2-yl)-1H-indole and 2-(pyridin-2-yl)-1H-pyrrolo[2,3-b]pyridine with BPh_3_ [36].

Bipyridine organoboron adducts have been synthesized using various boron reagents. Except for bipyridine as bidentate ligand, indole skeleton was chosen viably. In 2000, Suning Wang’s group has synthesized and characterized blue/green luminescent materials, diphenylboron complex (**38**, 65%), and complex (**40**, 71%) [36], as shown in Scheme 8. 2-(pyridin-2-yl)-1*H*-indole and 2-(pyridin-2-yl)-1*H*-pyrrolo[2,3-b]pyridine reacted with commercially available triphenylboron to prepare the target products. The complex (**40**) has been developed to function as the light-emitting layer.

### 3.2. 2-(Pyridin-2-yl)-1H-indole Derivatives Reacting with BPh_3_

**Scheme 9:**
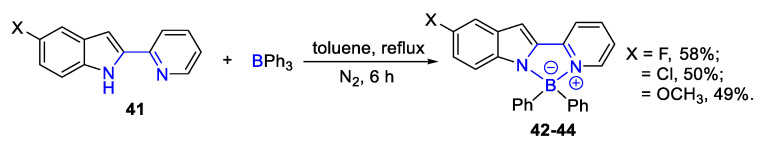
Reactions of 2-(pyridin-2-yl)-1H-indole derivatives with BPh_3_ [45].

In 2002, Suning Wang’s group explored more 5-substituted diphenylboron complexes. In this case, 5-fluoro-2-(pyridin-2-yl)-1*H*-indole, 5-chloro-2-(pyridin-2-yl)-1*H*-indole, and 5-methoxy-2-(pyridin-2-yl)-1*H*-indole could get desired products (**42**–**44**) [45], as shown in Scheme 9. They examined the effect of blue shift to 490 and 487 nm (**42**, **43**, -F, -Cl) and red shift to 532 nm (**44**, -OCH_3_), respectively. They found that compound **42** could be applied in an electroluminescent device as the emitter and the electron transport material. It was inferred that different substituted groups could tune the luminescence and the property of electroluminescence of *N*,*N*′-chelate organoboron compounds.

### 3.3. 2-(1H-Indol-2-yl)thiazole, 8-(1H-Benzo[d]imidazol-2-yl)Quinoline and Their Derivatives

**Scheme 10:**
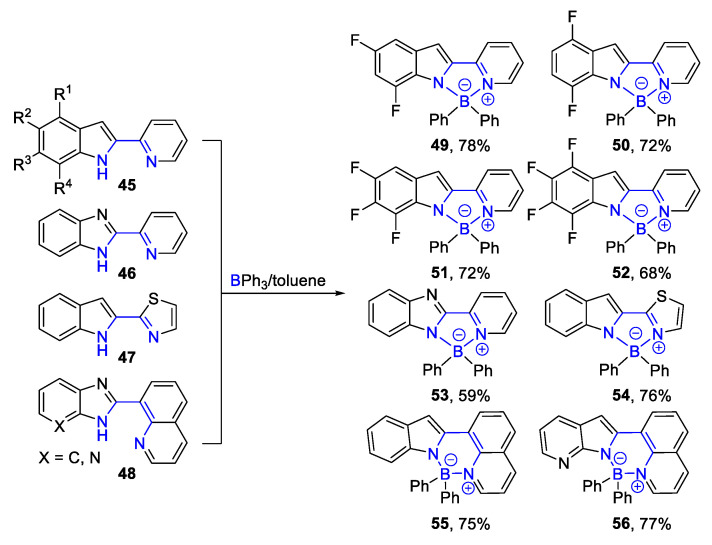
Reactions of 2-(1*H*-indol-2-yl)thiazole, 8-(1*H*-benzo[d]imidazol-2-yl)quinoline, and their analogues with BPh_3_ [30].

In 2005, Suning Wang’s group continued to present different indole skeleton bidentate ligands. Substituted-2-(pyridin-2-yl)-1*H*-indole (**45**), 2-(pyridin-2-yl)-1*H*-benzo[d]imidazole (**46**), 2-(1*H*-indol-2-yl)thiazole (**47**), 1-(1*H*-benzo[d]imidazol-2-yl)isoquinoline (**48**, X = C), 1-(3*H*-imidazo[4,5-b]pyridin-2-yl)isoquinoline (**48**, X = N) began with triphenylboron to generate *N*,*N*′ chelate boron complexes (**49**–**56**, 59–78%) from blue to red [30], as shown in Scheme 10. They also found that these complexes could be applied in not only emitters but also electron transport materials of electroluminescent (EL) equipment.

### 3.4. 2-(Pyridin-2-yl)-1H-indole Reacting with FcBMeBr

**Scheme 11:**
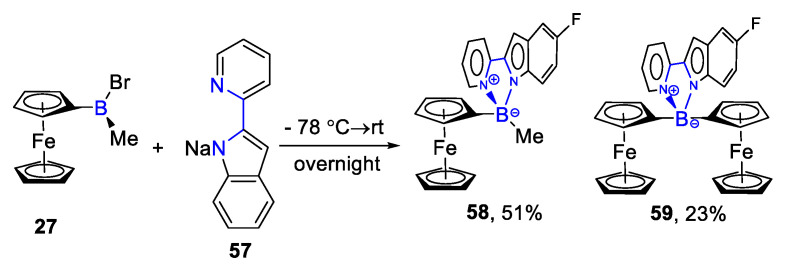
Reactions of 2-(pyridin-2-yl)-1H-indole with FcBMeBr [46].

In 2005, Matthias Wagner’s group achieved reaction of ferrocene bromide and 2-(pyridin-2-yl)-1*H*-indole sodium to give rise to the formation of FcBMe(5-F-bipyridine) (**58**, 51%) and Fc_2_B(5-F-bipyridine) (**59**, 23%) in acceptable yields [46], as shown in Scheme 11. It indicated that *N*,*N*′-chelate organoboron compounds containing multiple metals could be obtained by making use of this simple protocol.

### 3.5. 2-(1H-Indol-7-yl)benzo[d]oxazole, 2-(1H-Indol-7-yl)benzo[d]thiazole, and 2-(1H-Indol-7-yl)-1-octyl-1H-benzo[d]imidazole

**Scheme 12:**
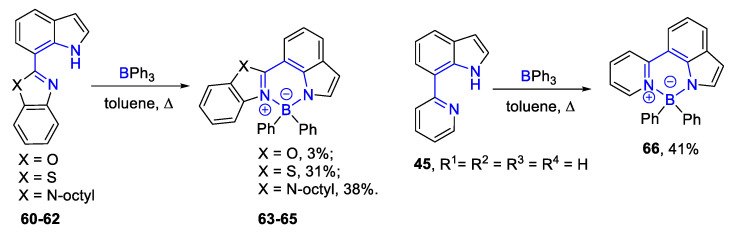
Reactions of 2-(1*H*-indol-7-yl)benzo[d]oxazole, 2-(1*H*-indol-7-yl)benzo[d]thiazole, and 2-(1*H*-indol-7-yl)-1-octyl-1*H*-benzo[d]imidazole with BPh_3_ [32].

In 2016, David Curiel’s group described a reaction of 2-(1*H*-indol-7-yl)benzo[d]oxazole (**60**), 2-(1*H*-indol-7-yl)benzo[d]thiazole (**61**), and 2-(1*H*-indol-7-yl)-1-octyl-1*H*-benzo[d]imidazole (**62**) to generate organoboron complexes (**63**–**65**) with tuned emission and cell bioimaging utility [32], as shown in Scheme 12. To synthesize these diphenylboron complexes, they choose triphenylboron as boron reagent. In this paper, they succeeded in introducing more heteroatoms (S, O, N) in this building block.

## 4. Pyridine-Based Derivatives as Bidentate Ligand

### 4.1. 2-(1H-Pyrazol-5-yl)pyridine Reacting with BPh_3_

**Scheme 13:**
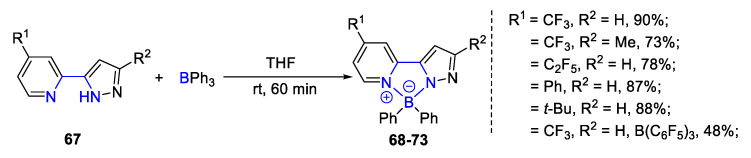
Reactions of 2-(1H-pyrazol-5-yl)pyridine reacting with BPh_3_ [47].

In 2003, Yun Chi’s group reported a reaction of 2-(1*H*-pyrazol-5-yl)pyridine derivatives (**67**) and triphenylboron to produce pyridyl pyrazolate boron complexes (**68**–**73**, 48–90%) [47], as shown in Scheme 13, which exhibited the ability of remarkable dual fluorescence through the photoinduced electron transfer reaction. In this system, it was more efficient to synthesize the *N*,*N*′-chelate organoboron adducts in tetrahydrofuran (THF) solution for one hour. They found that electron-deficient B(C_6_F_5_)_3_ reagent could be also feasible in this reaction.

### 4.2. Pyridin-2-ylmethanamine Reacting with Hydroxydiphenylborane

**Scheme 14:**
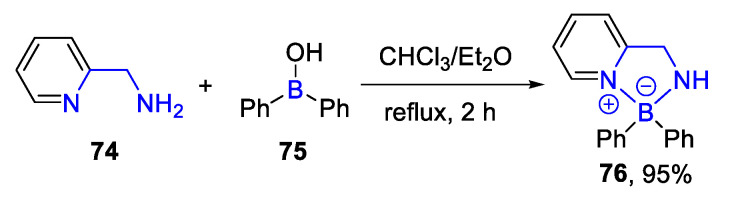
Reactions of pyridin-2-ylmethanamine with hydroxydiphenylborane [48].

In 2005, Noemí Andrade-López’s group provided another method to complete the *N*-8-(diphenyl-hydroxy-2-aminomethylpyridine)borane (**76**, 95%) with high yield when added 2-pyridylmethylamine (**74**) and diphenylborinic acid (**75**) together [48], as shown in Scheme 14. In this case, they used air-stable boron reagent (diphenylborinic acid). It was proved that nonrigid bidentate ligand 2-pyridylmethylamine could also be activated.

### 4.3. 2-(1H-Pyrazol-5-yl)pyridine Analogs Reacting with BPh_3_

**Scheme 15:**
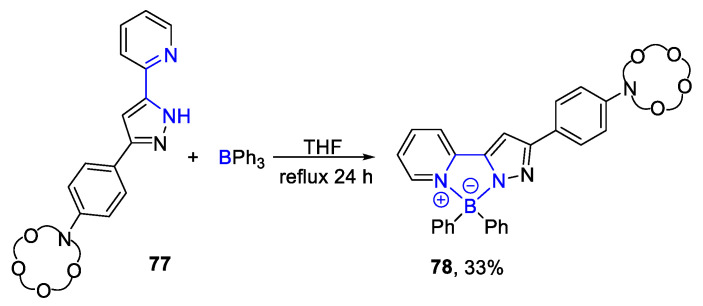
Reactions of 2-(1*H*-pyrazol-5-yl)pyridine derivatives with BPh_3_ [49].

In 2006, Yun Chi’s group developed 2-(1*H*-pyrazol-5-yl)pyridine derivatives (**77**) to acquire organoboron compound (**78**) [49], as shown in Scheme 15. It was interesting that the authors succeeded in introducing crown ether structure for detecting the alkali or alkaline earth ions. 

### 4.4. 2-(4H-1,2,4-Triazol-3-yl)pyridine Derivatives Reacting with BPh_3_

**Scheme 16:**
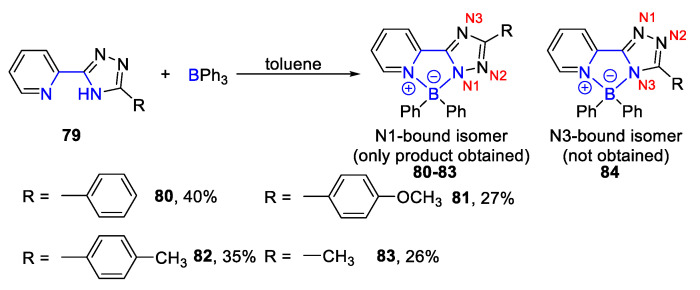
Reactions of 2-(4*H*-1,2,4-triazol-3-yl)pyridine derivatives with BPh_3_ [50].

In 2014, Wesley R. Browne’s group showed the resulting pyridyl-1,2,4-triazole tetracoordinate organoboron complexes as available and controllable blue emitters [50]. In this system, they could only obtain the N1-bound isomer (**80**–**83**), while N3-bound isomer was not found (**84**), as shown in Scheme 16. It was inferred that the boron atom of triphenylboron preferred to attacking with N1 atom center. The emission quantum yield of the complex (**80**) was up to 0.72. Synthesized compounds displayed a great range of emissions from 397 nm to 494 nm.

### 4.5. 2-(3,5-Bis(trifluoromethyl)-1H-pyrrol-2-yl)pyridine as Bidentate Ligand

**Scheme 17:**
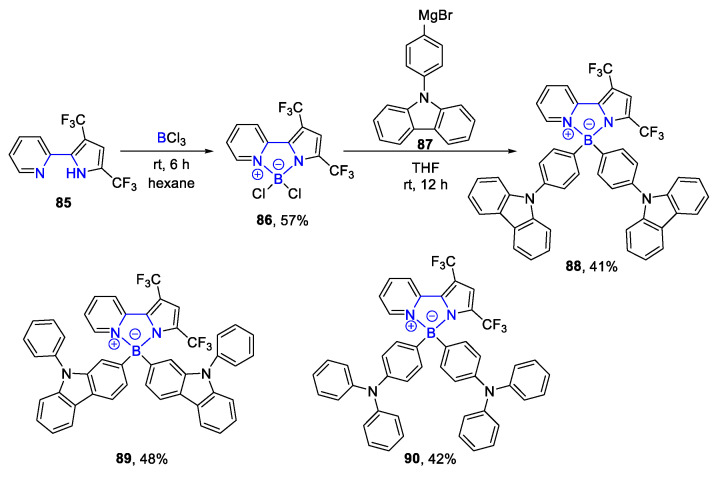
Synthesis of 2-(3,5-bis(trifluoromethyl)-1*H*-pyrrol-2-yl)pyridine chelate organoboron complexes [33].

In 2016, Yun Chi’s group provided a method for the synthesis of pyridyl pyrrolide boron complexes (**88**–**90**, 41–48%), which could be developed to function as organic light-emitting diodes (OLEDs) [33], as described in Scheme 17. This building block connected the electron-donating portion with the electron-accepting moieties together and succeed in generating external quantum efficiency (EQE) of 13.5%. 2-(3,5-bis(trifluoromethyl)-1*H*-pyrrol-2-yl)pyridine (**85**) and boron trichloride resulted in the intermediate compound (**86**, 57%). Next, it reacts with Grignard reagent (**87**) to give the final organoboron products (**88**–**90**). This protocol used the easy availability of Grignard reagent to produce pyridyl pyrrolide boron complexes.

### 4.6. 2-(5-Methyl-1H-pyrrol-2-yl)pyridine, 2-(Pyridin-2-yl)-1H-Indole, and (Z)-2-(Phenyl(2H-pyrrol-2-ylidene)methyl)-1H-pyrrole as Bidentate Ligand

**Scheme 18:**
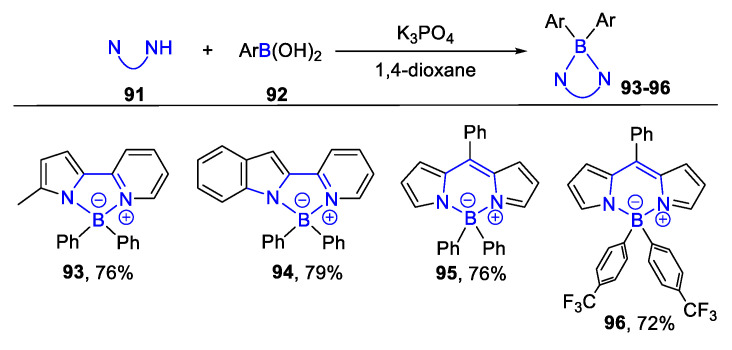
Reactions of 2-(5-methyl-1*H*-pyrrol-2-yl)pyridine, 2-(pyridin-2-yl)-1*H*-indole, and (*Z*)-2-(phenyl(2*H*-pyrrol-2-ylidene)methyl)-1*H*-pyrrole with BPh_3_ [51].

In 2017, Kee-In Lee’s group reported a synthetic route for the generation of *N*,*N*′-chelate organoboron complexes with mild condition and easy operation in moderate yields [51]. In this strategy, they added general phenylboronic acids to react with ligands using widely available K_3_PO_4_ to form desired products (**93**–**96**, 72–79%), as shown in Scheme 18. In this case, 2-(5-methyl-1*H*-pyrrol-2-yl)pyridine, 2-(pyridin-2-yl)-1*H*-indole, and (*Z*)-2-(phenyl(2*H*-pyrrol-2-ylidene)methyl)-1*H*-pyrrole could be able to respond smoothly.

## 5. Quinoline-Based Derivatives as Bidentate Ligand

### 5.1. 2-(5-Methyl-1H-pyrrol-2-yl)quinoline and 2-(5-Methyl-1H-pyrrol-2-yl)quinoxaline as Bidentate Ligand

**Scheme 19:**
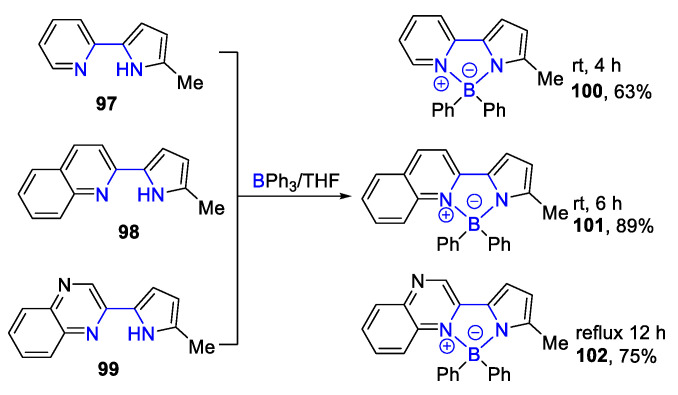
Reactions of 2-(5-methyl-1*H*-pyrrol-2-yl)pyridine, 2-(5-methyl-1*H*-pyrrol-2-yl)quinoline, and 2-(5-methyl-1*H*-pyrrol-2-yl)quinoxaline with BPh_3_ [52].

In 2005, Chin-Ti Chen’s group exploited 2-(5-methyl-1*H*-pyrrol-2-yl)pyridine (**97**), 2-(5-methyl-1*H*-pyrrol-2-yl)quinoline (**98**), and 2-(5-methyl-1*H*-pyrrol-2-yl)quinoxaline (**99**) reacting with triphenylboron to afford diphenylboron complexes (**100**–**102**, 63–89%) [52], as shown in Scheme 19. The complexes [(pyro)BPh_2_] (**100**), [(noro)BPh_2_] (**101**), and [(xaro)BPh_2_] (**102**) exhibited emission peak maxima of strong photoluminescence at different locations such as 490 nm, 510 nm and 575 nm, respectively. The research group adopted quinoline linked with pyrrole in one molecule as bidentate ligand to react with triphenylboron.

### 5.2. 2-(1H-Benzo[d]Imidazol-2-yl)quinoline and 2-(Quinolin-2-yl)-1H-naphtho[2,3-d]Imidazole Served as Bidentate Ligand

**Scheme 20:**
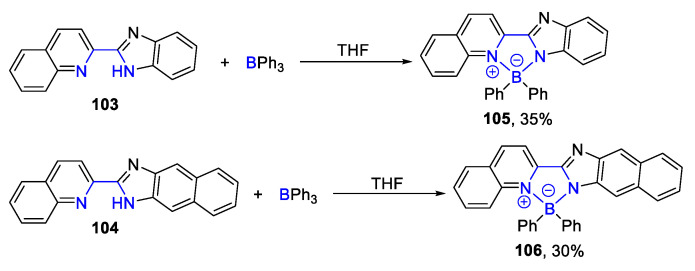
Synthesis of BPh_2_(2-(2-quinolyl)naphtho[b]imidazolato) and BPh_2_(2-(2-quinolyl)benzimidazolato) [53].

In 2006, Tsun-Ren Chen’s group realized 2-(1*H*-benzo[d]imidazol-2-yl)quinoline (**103**) and 2-(quinolin-2-yl)-1*H*-naphtho[2,3-d]imidazole (**104**) reacting with triphenylboron to generate diphenylboron complexes (**105**–**106**, 30–35%) [53], as shown in Scheme 20. The product (**105**) could be used as organometallic emitting material in organic light-emitting diodes (OLEDs). They succeeded in the synthesis of target organoboron products and adopted them (**105**, **106**) as the emitting materials of electroluminescence (EL) device. It was demonstrated that tetracoordinated organoboron complexes could be selected as organic light-emitting diodes (OLEDs) as well.

### 5.3. 1-(3-(Trifluoromethyl)-1H-pyrazol-5-yl)isoquinoline as Bidentate Ligand

**Scheme 21:**
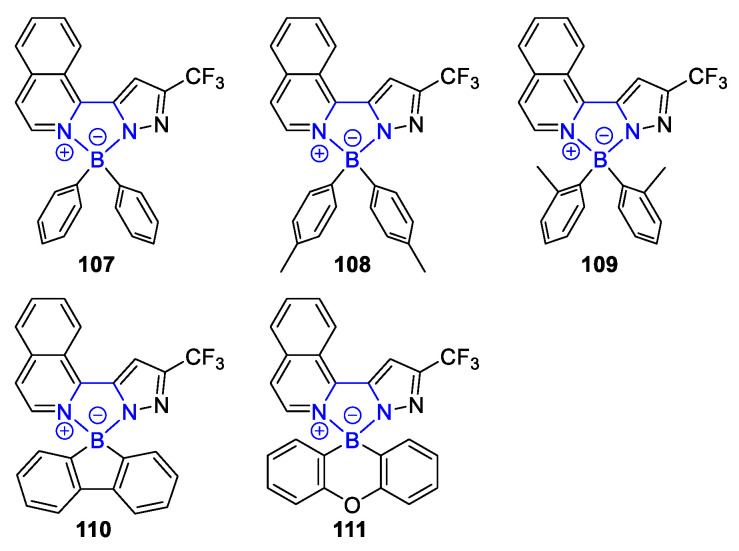
Synthesis of 1-(3-(trifluoromethyl)-1*H*-pyrazol-5-yl)isoquinoline chelate organoboron complexes [54].

Recently, in 2019, Pi-Tai Chou’s group designed and synthesized 1-isoquinolinyl pyrazolate biphenylboron complexes [54]. 1-(3-(trifluoromethyl)-1*H*-pyrazol-5-yl)isoquinoline was used to react with various boron reagents (BPh_3_, BCl_3_, 9-bromo-9-borafluorene, or 10-bromo-9-oxa-10-boraanthracene) to afford desired products (**107**–**111**), as shown in Scheme 21. The authors measured the time-resolved fluorescence and studied theoretical calculation, then found these complexes having the phenomenon of photoinduced electron transfer (PET) from the locally excited (LE) (over the isoquinolinyl pyrazolate moiety) to charge transfer (CT) states (from isoquinolinyl to aryl appendages).

### 5.4. N-(Quinolin-8-yl)acetamide as Bidentate Ligand

**Scheme 22:**
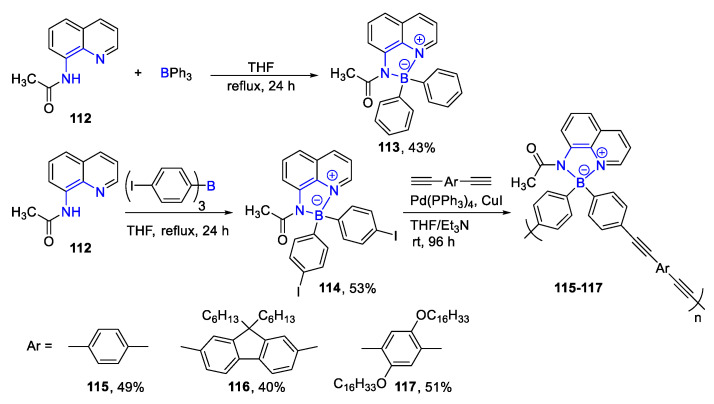
Reactions of *N*-(quinolin-8-yl)acetamide with BPh_3_ [34].

In 2008, Yoshiki Chujo’s group prepared *N*,*N*′-chelate tetracoordinated organoboron polymers by Sonogashira-Hagihara cross-coupling reaction [34]. They reported a reaction of *N*-(quinolin-8-yl)acetamide (**112**) and triphenylboron (43%), as shown in Scheme 22. The research group used tri(4-iodinephenyl)boron to synthesize the monomer (**114**, 53%). It continued to react with alkynes to obtain the final polymers (**115**–**117**, 40–51%). This paper showed that the π-conjugated linker unit had a great influence on the fluorescent quantum efficiencies of the synthesized polymers. Besides, they also observed an obvious energy transfer.

### 5.5. (S)-N-(1-((5-Iodoquinolin-8-yl)amino)-1-oxopropan-2-yl)hexanamide as Bidentate Ligand

**Scheme 23:**
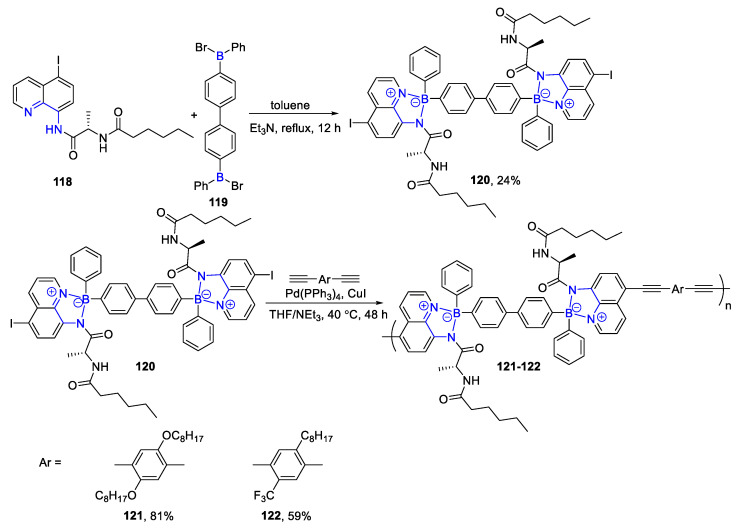
Synthesis of (S)-*N*-(1-((5-iodoquinolin-8-yl)amino)-1-oxopropan-2-yl)hexanamide chelate organoboron polymer [55].

In 2010, Yoshiki Chujo’s group used (S)-*N*-(1-((5-iodoquinolin-8-yl)amino)-1-oxopropan-2-yl)hexanamide (**118**) and diphenyl with two boronbromide centers resulting in organoboron monomer (**120**, 24%) [55], as shown in Scheme 23. They chose palladium-catalyzed Sonogashira coupling between iodide (**120**) and bis-alkyne to prepare luminescent chiral four-coordination organoboron quinoline-based polymers. It was interesting to see that the quantum yield (Φ_F_) of the synthesized polymer (**122**) was up to 0.8.

### 5.6. N-(5-Iodoquinolin-8-yl)undecanamide as Bidentate Ligand

**Scheme 24:**
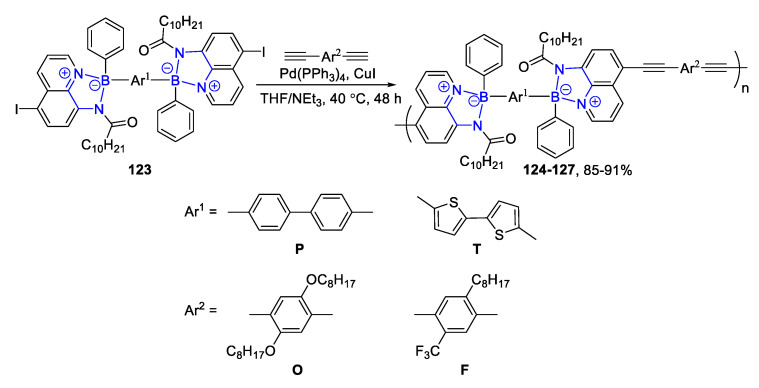
Synthesis of *N*-(5-iodoquinolin-8-yl)undecanamide chelate organoboron polymer [56].

In 2010, Yoshiki Chujo’s group developed strong luminescent organoboron polymers connected with bifunctional 8-aminoquinolate linkers [56]. They used *N*-(5-iodoquinolin-8-yl)undecanamide and bis-alkyne resulting in corresponding polymers (**124**–**127**) by Sonogashira-Hagihara cross-coupling reaction, as shown in Scheme 24. In this case, they introduced long alkyl chains and various aryl groups and finally found the organoboron polymer (**127**, Ar^1^ = P, Ar^2^ = F, 85%) (Φ_F_ = 0.65) achieving a higher quantum yield than others. Compared with (*S*)-*N*-(1-((5-iodoquinolin-8-yl)amino)-1-oxopropan-2-yl)hexanamide as starting material to obtain *N*,*N*′-chelate organoboron complexes, *N*-(5-iodoquinolin-8-yl)undecanamide as bidentate ligand showed different optical properties. It indicated that the fluorescent characteristic of organoboron polymers could be changed by different substituted portions.

### 5.7. N-(quinolin-8-yl)acetamide as Bidentate Ligand

**Scheme 25:**
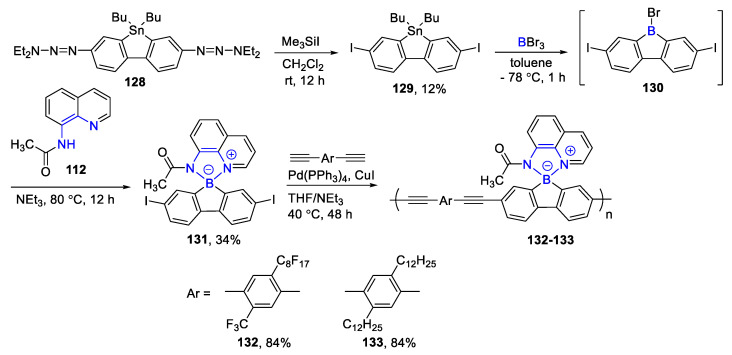
Synthesis of *N*-(quinolin-8-yl)acetamide chelate organoboron polymer [57].

Two years later, in 2012, Yoshiki Chujo’s group designed and synthesized 8-aminoquinolate-based organoboron polymers with rigid structure, which had electron-donating and electron-accepting structure in the basic skeleton [57]. In order to get the corresponding starting material, they added tin compound (**128**) and boron tribromide to give the intermediate (**130**) in two steps, as shown in Scheme 25. Next, it continued to react with *N*-(quinolin-8-yl)acetamide to afford adduct monomer in acceptable yield (**131**, 34%). Final 8-aminoquinolate-based organoboron products were prepared by classical Sonogashira-Hagihara cross-coupling reaction (**132**, **133**, 84%, 84%). The quantum yield of polymer (**132**) was up to 0.53. It was certain that this scaffold with 8-aminoquinolate-based could achieve more efficient luminescent properties by introducing the -C_8_F_17_ group.

### 5.8. Quinolin-8-amine as Bidentate Ligand

**Scheme 26:**
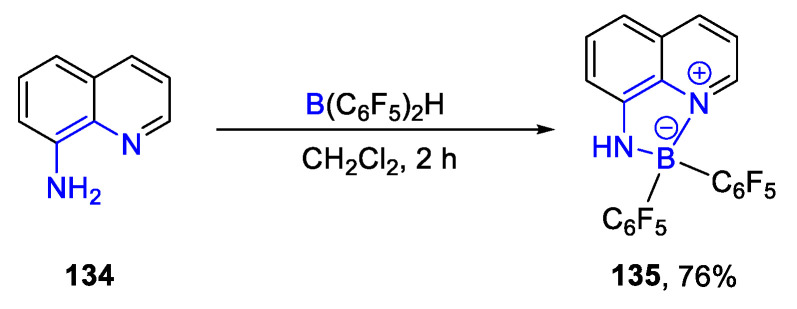
Synthesis of quinolin-8-amine chelate organoboron complex [58].

In 2012, Douglas W. Stephan’s group reported a reaction of readily available quinoline-8-amine (**134**) and electron-deficient B(C_6_F_5_)_2_H [58]. Biperfluorophenylboron complex was synthesized in moderate yield (**135**, 76%) without any catalyst, as shown in Scheme 26. The result indicated that tetracoordinated organoboron compounds could also be successfully obtained from primary amines.

### 5.9. 3-Phenyl-N-(quinolin-8-yl)propanamide Derivatives as Bidentate Ligand

**Scheme 27:**
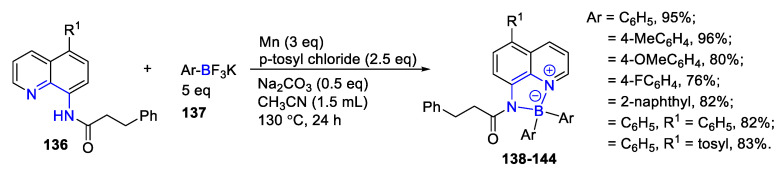
Synthesis of 3-phenyl-*N*-(quinolin-8-yl)propanamide derivatives chelate organoboron photocatalysts [35].

In 2020, Xu’s group designed and synthesized a series of 3-phenyl-*N*-(quinolin-8-yl)propanamide-based organoboron complexes as photocatalysts [35]. At first, they prepared starting materials 3-phenyl-*N*-(quinolin-8-yl)propanamide derivatives (**136**) that were added in Mn/p-tosyl chloride/Na_2_CO_3_ system. Desired 3-phenyl-*N*-(quinolin-8-yl)propanamide-based organoboron complexes were acquired with high yields (**138**–**144**, 80–96%), as shown in Scheme 27. In this case, they added cheap transitional metal manganese powder. It was worth noting that they used widely available and air-stable boron reagents (Ar-BF_3_K, **137**). This protocol provided a useful path for the generation of aminoquinolate-based organoboron complexes efficiently.

### 5.10. 2,2,2-Trifluoro-N-(quinolin-8-yl)acetamide Derivatives as Bidentate Ligand

**Scheme 28:**
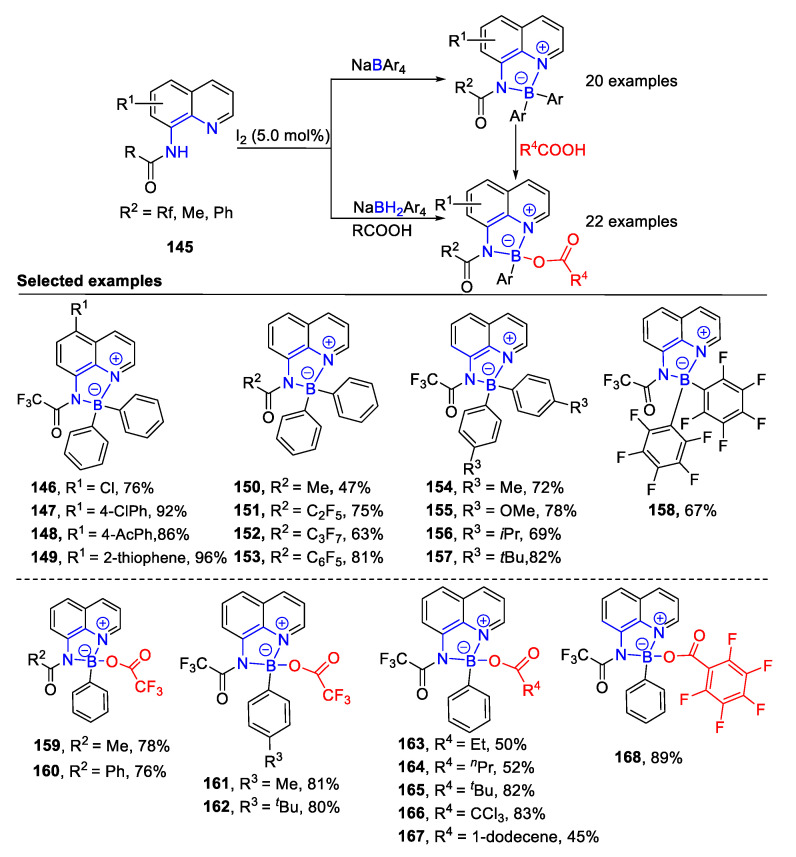
Synthesis of 2,2,2-trifluoro-*N*-(quinolin-8-yl)acetamide-based organoboron fluorescent complexes [31].

In 2020, our group almost at the same time reported the reaction of 2,2,2-trifluoro-*N*-(quinolin-8-yl)acetamide derivatives (**145**) and sodium tetraarylborate, resulting in aminoquinolate-based organoboron complexes (**146**–**168**) with high yields using iodine as catalyst [31], as depicted in Scheme 28. We made a modification from 2,2,2-trifluoro-*N*-(quinolin-8-yl)acetamide with various functional groups. In our protocol, we synthesized successfully a series of cheap and air-stable sodium tetraarylborates. Only a catalytic amount of iodine was enough to drive this reaction. In addition, we showed that adding external acids in this system formed the novel *N*,*N*′-chelate organoboron aminoquinolate with moderate yields (**159**–**168**, 45–89%). All the complexes were fully characterized and we found that the quantum yield of organoboron compound (**146**) reached 0.79 when dissolved in dichloromethane.

## 6. 1,10-Phenanthroline and Others as Bidentate Ligand

### 6.1. 1,10-Phenanthroline as Bidentate Ligand

**Scheme 29:**
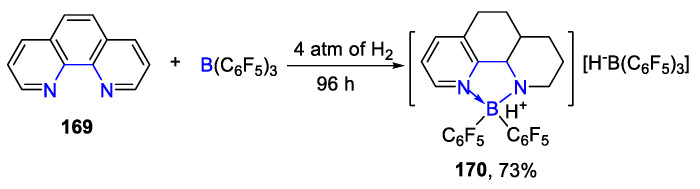
Synthesis of 1,10-phenanthroline chelate organoboron complex [59].

In 2013, Douglas W. Stephan’s group treated 1,10-phenanthroline with the same amount of the boron reagent B(C_6_F_5_)_3_ to afford the desired salt (**170**, 73%) under H_2_ (4 atm) condition [59], as shown in Scheme 29. The authors tried to develop an organoboron catalyst, which has the ability of frustrated Lewis pairs (FLPs) catalyst in practical applications. In this paper, they developed a new synthetic route of tetracoordinated organoboron complexes by using 1,10-phenanthroline.

### 6.2. 1,10-Phenanthroline as Bidentate Ligand

**Scheme 30:**
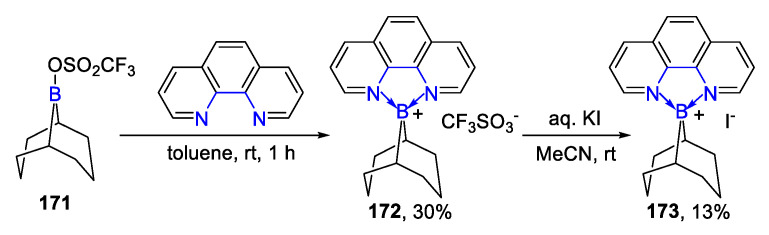
Synthesis of 1,10-phenanthroline chelate organoboron complex [60].

In 2016, Hiroyuki Higuchi’s group also reported 1,10-phenanthroline as bidentate ligand to react with 9-borabicyclo[3.3.1]nonan-9-yl trifluoromethanesulfonate to obtain the organoboron complex (**172**, 30%) [60], as shown in Scheme 30. Next, it was reacted with potassium iodide solution to afford the corresponding salt (**173**, 13%). It was noteworthy that the synthesized complex (**173**) displayed photoinduced different color behavior in the solid-state after UV irradiation.

### 6.3. (9E,10E)-N^9^,N^10^-Bis(6-methylheptyl)phenanthrene-9,10-diimine as Bitentate Ligand

**Scheme 31:**
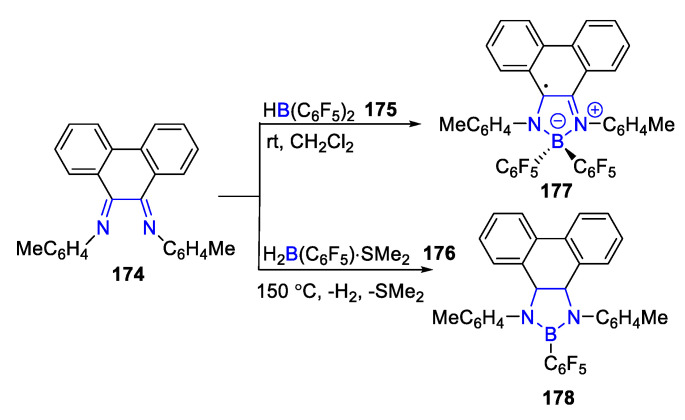
Reactions of (9E,10E)-N^9^,N^10^-bis(6-methylheptyl)phenanthrene-9,10-diimine with trivalent boron [61].

In 2017, Douglas W. Stephan’s group described an original synthetic strategy to give rise to (9E,10E)-N^9^,N^10^-bis(6-methylheptyl)phenanthrene-9,10-diimine-based organoboron complex (**177**), but only found the radical (**177**) measured by direct analysis in real time mass spectrometry (DART-MS) and electron paramagnetic resonance (EPR) signal [61], as shown in Scheme 31. They continued to improve and develop previous work and used HB(C_6_F_5_)_2_ reagent to react with bidentate ligand (**174**) to prepare frustrated Lewis pairs (FLPs) catalyst in this paper.

### 6.4. Diketopyrrolopyrrole (DPP) Derivatives as Bitentate Ligand

**Scheme 32:**
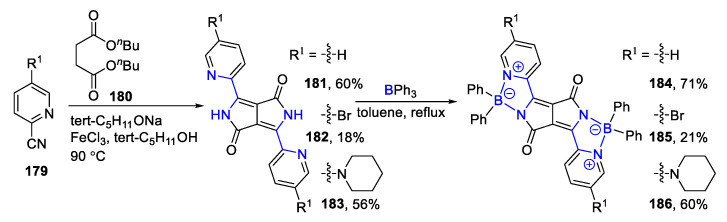
Synthesis of boron-coordinated diketopyrrolopyrrole (DPP) derivatives [62].

In 2014, Takaki Kanbara and co-workers disclosed a route for the generation of diketopyrrolopyrrole (DPP)-based diphenylboron complexes with moderate yields (**184**–**186**, 21–71%) [62], as shown in Scheme 32. In this case, diketopyrrolopyrrole derivatives were synthesized by using 5-substituted-picolinonitrile (**179**) and dibutyl succinate (**180**). Next, triphenylboron reacted with them to form the diphenylboron complexes (**184**–**186**). At last, the authors studied the corresponding theoretical calculation in their work, which indicated that the introduction of the coordinating boron element could lower the band gap.

### 6.5. Diketopyrrolopyrrole (DPP) Derivatives as Bitentate Ligand

**Scheme 33:**
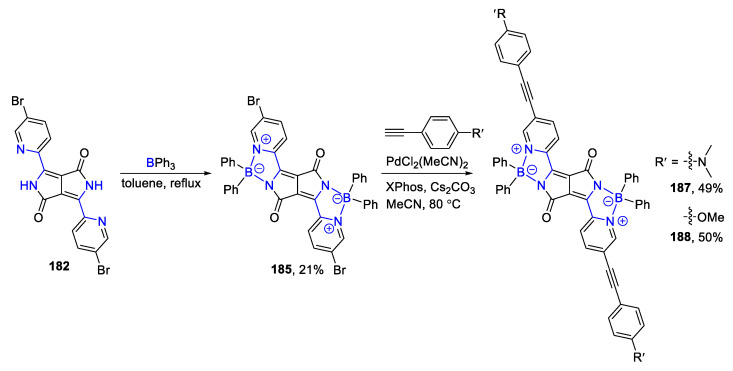
Synthesis of boron-coordinated diketopyrrolopyrrole (DPP) derivatives [63].

In 2014, Takaki Kanbara and co-workers continued to improve this scaffold by introducing phenylacetylene derivatives [63], by palladium-catalyzed Sonogashira coupling. Synthesized diketopyrrolopyrrole-based diphenylboron complexes (**187**–**188**) showed red-shift of the location of wavelengths to a near-infrared region, as shown in Scheme 33. This was a good strategy to extend π-conjugation of diketopyrrolopyrrole-based diphenylboron complexes by linking with more rigid phenylethynyl groups, which reached red-shift in near-infrared region and achieved high quantum yields.

### 6.6. Reactivity of 1,2,3,4,5-Pentaphenyl-1H-borole with Isocyanates

**Scheme 34:**
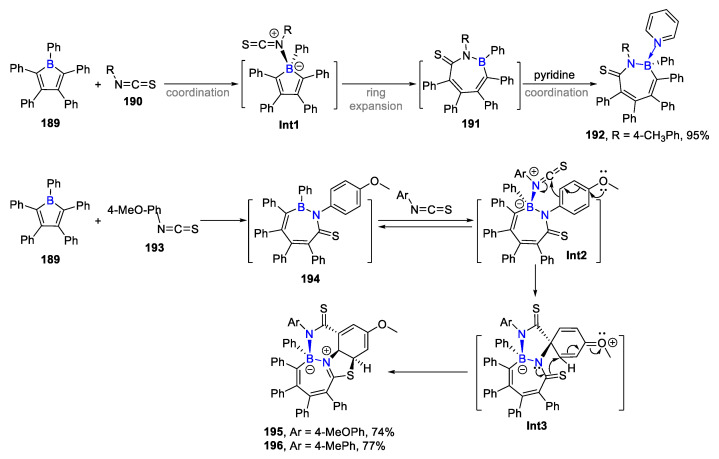
Reactions of 1,2,3,4,5-pentaphenyl-1*H*-borole with isocyanates [64].

In 2016, Caleb D. Martin’s group used 1,2,3,4,5-pentaphenyl-1*H*-borole (**189**) with isocyanates to generate a seven-membered ring (**194**) and it continued to react with another isocyanate to afford more complex organoboron products (**195**, **196**, 74%, 77%, respectively) [64], as shown in Scheme 34. The boron atom of the complex (**191**) would attack the nitrogen atom in the presence of pyridine to form a new adduct (**192**, 95%).

## 7. Conclusions and Outlook

In summary, tetracoordinated organoboron compounds have been widely applied in cell imaging, organic light-emitting diodes (OLEDs), functional polymer, photocatalyst, electroluminescent (EL) devices, and other science and technology areas. *N*,*N*′-chelate diarylboron complexes have been successfully synthesized by using a series of bidentate ligands and diversified boron reagents. Bidentate ligands mainly consist of two parts by using bipyridine, indole, pyrrole, quinoline, isoquinoline, pyridine, 1*H*-benzo[d]imidazole, and others. The boron reagents mainly include triphenylboron, boron trichloride, boron tribromide, B(C_6_F_5_)_3_, HB(C_6_F_5_)_2_, hydroxydiphenylborane (Ph_2_BOH), phenylboronic acids, sodium tetraaylborate, and FcMeBr in this review. However, there is main focus including exploring wider substrates as bidentate ligand, more diversified boron reagents, simpler and greener reaction condition, and higher yields. Besides, this *N*,*N*′-chelate organoboron framework should be developed in these fields such as molecular probes, cell imaging, fluorescent dyes, OLEDs, and so on. We expect with continuous effort more efficient and greener methods for exploiting more powerful diphenylboron complexes.

## Data Availability

The data presented in this study are openly available in references [1,2,3,4,5,6,7,8,9,10,11,12,13,14,15,16,17,18,19,20,21,22,23,24,25,26,27,28,29,30,31,32,33,34,35,36,37,38,39,40,41,42,43,44,45,46,47,48,49,50,51,52,53,54,55,56,57,58,59,60,61,62,63,64].
